# Multidisciplinary Oral Rehabilitation in Osteogenesis Imperfecta: 18‐Year‐Old Case Report

**DOI:** 10.1002/ccr3.73383

**Published:** 2026-08-24

**Authors:** Pegah Mosannen Mozafari, Mahsa Talafi Noghani, Iman Kashani, Reza Beyragh

**Affiliations:** ^1^ Shahab Dental Clinic (Oral Medicine and Special Care Dentistry) Mashhad Iran; ^2^ Faculty of Dentistry Mashhad University of Medical Sciences Mashhad Iran; ^3^ Pediatric Anesthesia, Department of Pediatrics, Faculty of Medicine Mashhad University of Medical Sciences Mashhad Iran; ^4^ Private Dental Practice Mashhad Iran

**Keywords:** case report, conservative dental management, failure of tooth eruption, general anesthesia, osteogenesis imperfecta

## Abstract

Osteogenesis imperfecta (OI) is a hereditary connective tissue disorder characterized by bone fragility and Type I collagen defects. Although dentinogenesis imperfecta (DI) is a classic manifestation, patients with OI may experience significant dental deterioration even in its absence due to inherent dentinal weakness. This report describes the multidisciplinary full‐mouth rehabilitation of a 18‐year‐old male with OI and a complex medical history including autoimmune hepatitis and seizure disorder. The patient presented with extensive caries, failure of previous restorations, multiple impacted teeth, and radiographic evidence of preeruptive intracoronal resorption (PEIR) as well as open apices in permanent second molars. Severe microstomia, joint laxity, high risk of iatrogenic jaw fracture, and limited cooperation necessitated treatment under general anesthesia. Management included vital pulp therapy to promote apexogenesis in immature teeth, stainless steel crowns for posterior rehabilitation, and composite restorations using universal adhesive systems containing 10‐MDP. A conservative, non‐extraction strategy was adopted to minimize mandibular fracture risk. This case highlights that dental pathology in OI may originate within bone prior to eruption and emphasizes the importance of biologically driven and fracture‐conscious treatment planning in medically complex patients.

## Key Clinical Message

Dental deterioration in osteogenesis imperfecta may begin intraosseously, independent of oral environmental factors. Comprehensive rehabilitation under general anesthesia prioritizes fracture prevention over behavior control. Biologically based therapies such as vital pulp therapy, combined with advanced adhesive systems, are essential for preserving structurally compromised dentition.

## 1 Introduction

Osteogenesis imperfecta (OI) is a rare heritable connective tissue disorder characterized by bone fragility, recurrent fractures, and skeletal deformities. It primarily results from mutations in the COL1A1 or COL1A2 genes, leading to quantitative or qualitative defects in Type I collagen. Beyond skeletal involvement, extraskeletal manifestations such as blue sclerae, hearing loss, and joint laxity often necessitate early multidisciplinary intervention [1, 2, 3]. The clinical spectrum of OI is highly variable, ranging from mild forms to progressively deforming and perinatally lethal forms [4, 5].

Dentinogenesis imperfecta (DI) represents a common hallmark; however, other anomalies, including increased susceptibility to dental caries, tooth agenesis (14%–24%), and tooth impactions—particularly of second permanent molars (33%)—are also frequently reported in OI patients [2, 3]. Pediatric dental management therefore emphasizes preventive strategies and the use of stainless steel crowns or composite restorations to protect structurally compromised teeth [1, 6, 7]. Additionally, bisphosphonate therapy, which is commonly prescribed in children with OI, requires meticulous coordination for surgical procedures to minimize the risk of delayed and deficient healing [1, 3, 8].

This study presents a case of OI with multiple impacted and missing teeth in the absence of dentinogenesis imperfecta, highlighting a comprehensive treatment approach.

## 2 Case History

A 15‐year‐old male patient was referred to the Shahab Dental Clinic (Special Care and Oral Medicine Clinic) in March 2022 with the chief complaint of extensive dental caries and a request for comprehensive restorative treatment under general anesthesia (GA) due to lack of cooperation.

The patient had a confirmed diagnosis of OI, and his family history was notable for consanguineous parentage, which can be associated with specific autosomal recessive forms of OI. His medical history indicated severe extraskeletal involvement, including autoimmune hepatitis, hepatic fibrosis, and inflammatory bowel disease (IBD). Additionally, his parents reported a history of recurrent seizures and previous blood product transfusions. Clinical examinations revealed limited mouth opening, possibly due to microstomia and underlying skeletal defects.

The patient was managed with an extensive pharmacological regimen that included Ursodeoxycholic acid (Ursaflor) and Esomeprazole (Nexium). Neurological and anticonvulsant therapy consisted of Levetiracetam (Levebel), Clobazam, Topiramate, Vigabatrin (Sabril), and Risperidone. Additional medications and supplements included Acetazolamide and vitamins A, D, E, and K. The patient had not received bisphosphonates for treatment of OI.

## 3 Examination and Diagnosis

**FIGURE 1:**
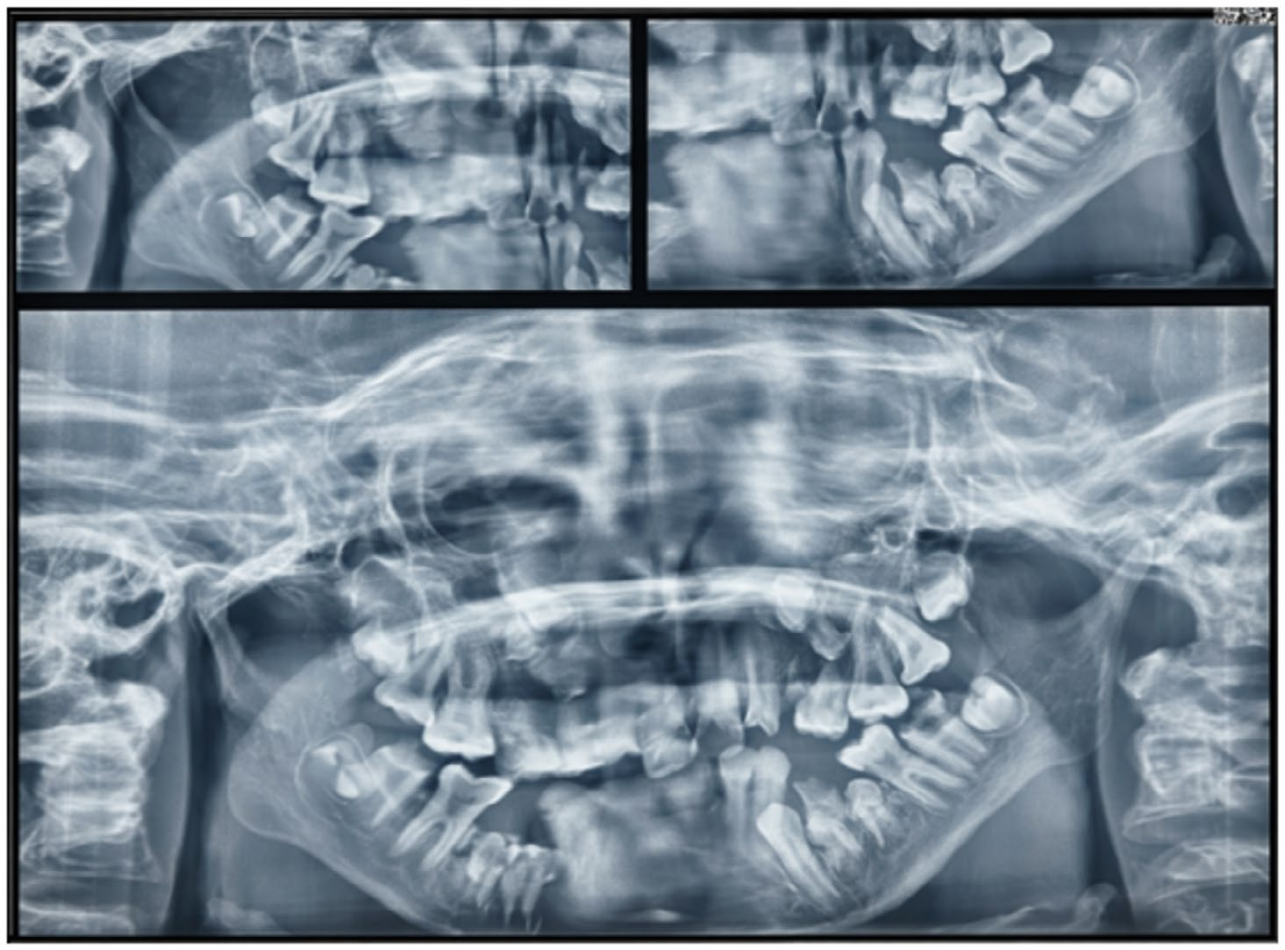
Initial panoramic radiograph (OPG) obtained in 2021 (1400 SH).

Baseline panoramic radiography demonstrated the initial extent of dental caries and the patient's overall oral condition at the time of first presentation, serving as a reference for subsequent disease progression (Figure 1). Although a comprehensive treatment plan was established in 2022, the patient did not comply with the appointments for approximately three years. During this period, he received dental treatment at another private facility.

Upon re‐evaluation in 2025, the patient's systemic condition had deteriorated significantly. Notably, the development of splenomegaly resulted in secondary thrombocytopenia, further increasing the complexity of dental management. The patient returned to the clinic for definitive oral rehabilitation to emend previous procedural shortcomings and address current dental problems. At the time of reassessment, the patient was fully dependent on parents for oral hygiene, and overall oral health status was poor.

Clinical examinations in 2025 were not possible due to the patient's lack of cooperation. Consequently, an initial assessment was performed using video graphic documentation, and a comprehensive intraoral re‐examination was subsequently conducted under GA at the beginning of the surgery. Examinations revealed a significant failure rate of previous dental treatments, primarily characterized by the breakdown of composite restorations and the presence of extensive recurrent carious lesions. The patient exhibited severely compromised oral hygiene, which directly contributed to the rapid progression of extensive carious lesions.

**FIGURE 2:**
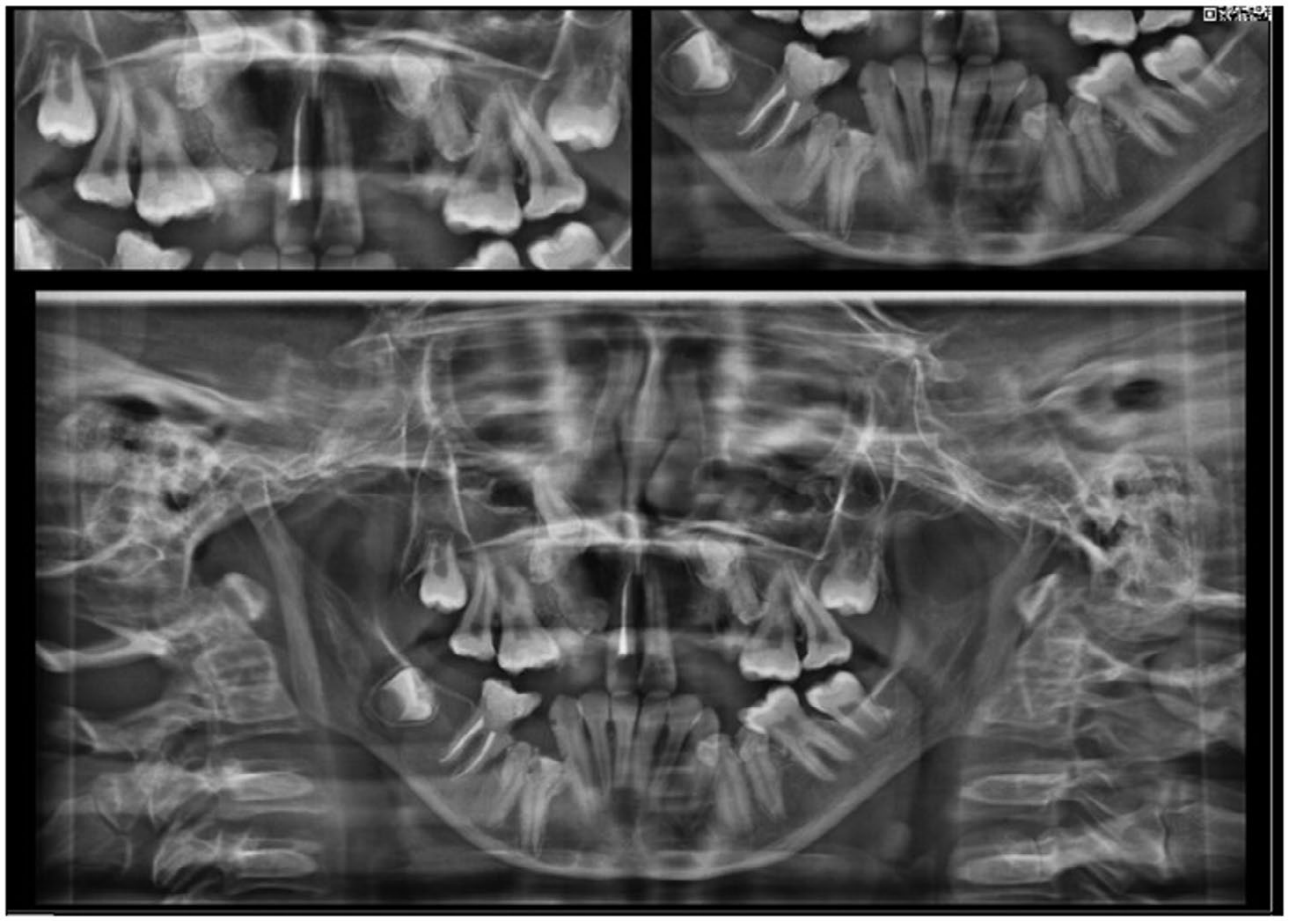
Preoperative panoramic radiograph (OPG) obtained in 2025 (1404 SH) revealing severe clinical deterioration.

**FIGURE 3:**
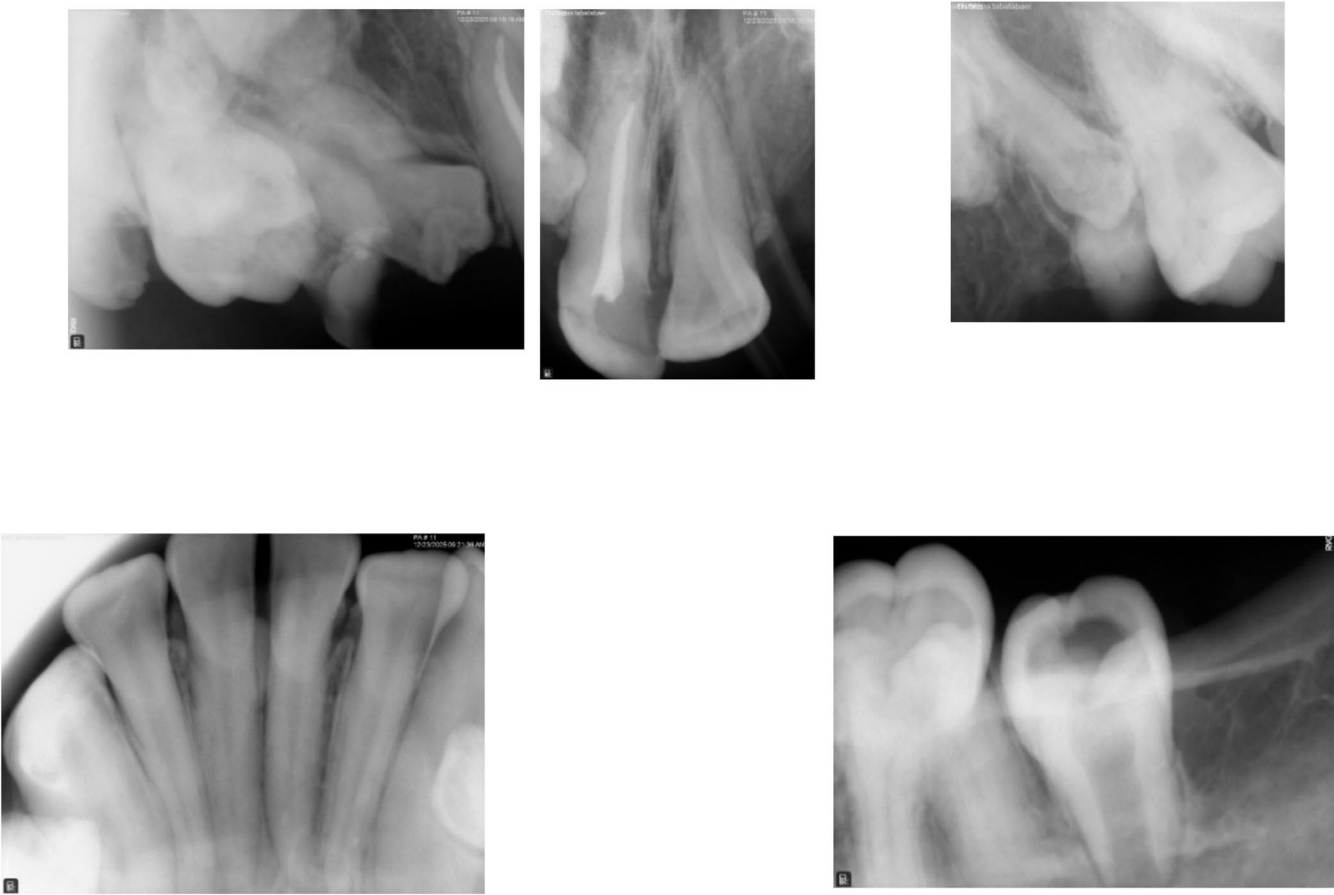
Preoperative intraoral periapical radiographs obtained in 2025 (1404 SH).

Comprehensive radiographic assessment using panoramic imaging (OPG) in 2025 revealed a complex spectrum of dental pathologies and developmental disorders (Figures 2 and 3), as detailed below:

- Radiographic examinations demonstrated extensive, deep carious lesions in teeth #17, #27, and #37, with radiolucencies in close proximity of the pulp chambers. On the other hand, teeth #36 and #43 exhibited moderate‐depth carious lesions without any evidence of pulpal involvement. Notably, several previously restored teeth with composite resin (#16, #26, #46, #11, and #21) showed extensive recurrent caries beneath existing restorations, underscoring the susceptibility of dental tissues in patients with OI. Additionally, retained roots of the maxillary primary second molars (#55 and #65) were identified.
- A rare developmental finding consistent with pre‐eruptive intracoronal resorption (PEIR), radiographically resembling caries, was detected within the crowns of impacted teeth #44 and #45 prior to their eruption into the oral cavity.
- Noticeable disruptions in teeth eruption were evident. Teeth #13, #14, #15, #23, #24, #25, #34, #35, #44, and #45 remained impacted within the alveolar bone. This extensive pattern of impaction, particularly involving canines and premolars, is a well‐recognized phenotypic manifestation associated with OI.
- Among the unerupted teeth, the maxillary premolars (#14, #15, #24, and #25) showed distinct morphological abnormalities involving both crown and root structures.

## 4 Treatment

### 4.1 Anesthesia and Surgical Precautions

The extensive dental treatment requirements, combined with the patient's systemic complexity, necessitated a comprehensive full‐mouth rehabilitation under general anesthesia. The proposed treatment plan was thoroughly discussed with the patient's legal caregivers, and written informed consent was obtained prior to surgery.

- Preoperative Assessment: Given the multisystem involvement characteristic of OI, a detailed preoperative evaluation was undertaken to assess risks associated with skeletal deformities, including scoliosis and thoracic cage abnormalities, as well as potential cardiopulmonary insufficiency. This assessment was essential to minimize perioperative respiratory complications, which remain a leading cause of morbidity and mortality in patients with OI.
- Airway Management: Airway management and intubation posed significant challenges due to anatomical features commonly associated with OI, including microstomia, a short neck, restricted cervical mobility, macroglossia, and craniofacial anomalies. To reduce the risk of cervical spine injury during airway manipulation and patient positioning, endotracheal intubation was performed with extreme caution using a video laryngoscope, allowing precise visualization and controlled instrumentation.
- Positioning and Handling (IV Access Vigilance): To minimize the risk of iatrogenic fractures during patient transfer and throughout the surgical procedure, extensive protective measures were implemented, including the use of padding, pillows, and specialized sandbags to provide full support for the limbs and spine. Particular vigilance was exercised during the establishment of intravenous (IV) access. According to the patient's father, the patient had sustained two bone fractures during previous IV insertion attempts. Consequently, following inhalational induction with Sevoflurane, all procedures involving external pressure—including tourniquet application and cannula stabilization—were performed with maximal gentleness and under protective cushioning.
- Joint and bone Protection: Given the combination of ligamentous laxity and extreme bone fragility associated with OI, mouth opening was achieved using minimal and carefully controlled forces. This precaution was essential to prevent temporomandibular joint (TMJ) dislocation as well as iatrogenic mandibular fractures, particularly in the vulnerable condylar neck region.
- Hemorrhage Control and Systemic Monitoring: Given the inherent capillary and tissue fragility in OI, compounded in this patient by secondary thrombocytopenia, the surgical and anesthetic teams maintained full preparedness for intraoperative hemorrhage management. Continuous monitoring of vital signs—including electrocardiography (ECG), capnography, noninvasive blood pressure, heart rate, and oxygen saturation—was strictly enforced due to the elevated risk of respiratory compromise throughout the procedure. As a prophylactic measure to reduce the bleeding risk associated with thrombocytopenia, tranexamic acid was administered intravenously at a dose of 10 mg/kg prior to tooth extraction (Remaining roots of the maxillary primary molars were extracted with minimal trauma).

### 4.2 Restorative and Endodontic Interventions

Comprehensive oral rehabilitation was performed as follows:

- Posterior Teeth (#17, #27, #37) were treated with vital pulp therapy (VPT) and subsequently restored with stainless steel crowns (SSCs) to provide full coverage and protect against further structural wear, caries, and fractures.
- For teeth with recurrent caries (#16, #26), existing failing composite restorations were removed and replaced with SSCs to ensure durable and caries‐resistant coverage.
- For tooth #46, following complete removal of recurrent caries, the buccal margin was subgingival, and to achieve an adequate marginal seal in an area unsuitable for direct crown margin placement, the tooth was initially restored with dental amalgam and subsequently covered with an SSC.
- Tooth #36 received a preventive resin restoration (PRR), and after a thorough caries removal, the restorations of anterior teeth (#11 and #12) were replaced.

### 4.3 Conservative Management of Impacted Teeth

Initial radiographic evaluation of the impacted teeth suggested the presence of internal defects. However, subsequent clinical examinations confirmed that the affected teeth were structurally sound. Tooth #34 was partially exposed, with approximately one‐third of the crown visible intraorally, and exhibited a surface appearance mimicking clinical caries.

A conservative, non‐extraction approach was adopted for all impacted teeth. This management strategy was justified by the substantial risk of mandibular fracture associated with surgical extraction in patients with osteogenesis imperfecta, as well as radiographic examinations suggesting ankylosis, indicated by complete root formation in the absence of a distinguishable periodontal ligament (PDL) space. Therefore, priority was given to radiographic and clinical monitoring of eruption patterns, along with reinforced oral hygiene instructions, rather than pursuing invasive surgical intervention.

## 5 Postoperative Management

For postoperative analgesia, paracetamol was administered at a dose of 15 mg/kg at the conclusion of the procedure. Emergence from anesthesia was smooth, and following a 30‐min recovery period, the patient was returned calmly to his parents.

**FIGURE 4:**
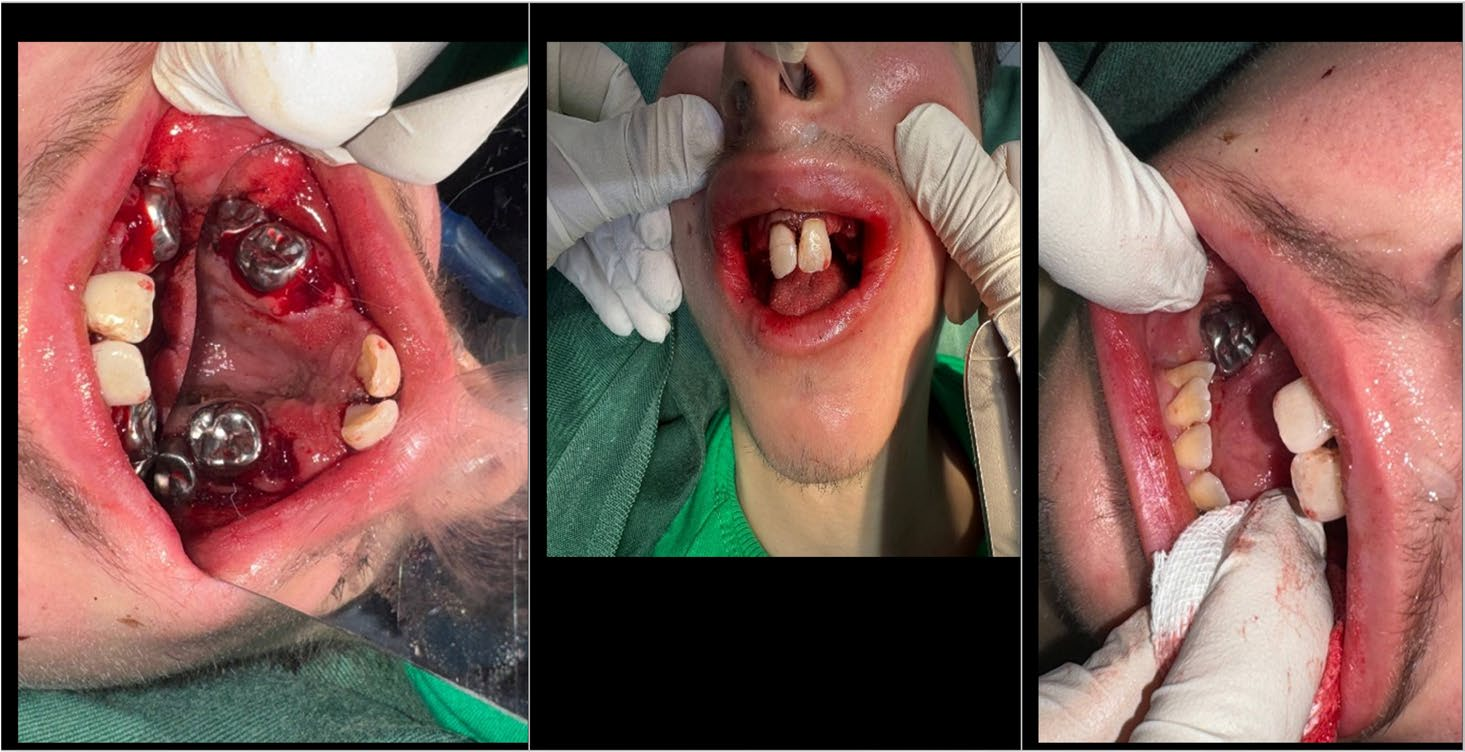
Postoperative photographs demonstrating the clinical outcomes after the intervention.

Postoperatively, systemic antibiotics were prescribed, and a strict follow‐up and preventive care protocol was established. Regular follow‐up appointments were scheduled, and comprehensive oral hygiene and oral health maintenance instructions were provided to the patient's parents. Figure 4 presents the postoperative photographs, illustrating the clinical outcomes following the intervention.

## 6 Discussion

OI results from defects in Type I collagen synthesis, which directly compromise the structural integrity of the dentin matrix [1, 3, 6]. In the present case, the rapid progression of extensive carious lesions in teeth #17, #27, and #37 underscores the inherent structural vulnerability of teeth affected by OI [6, 7, 9]. Notably, no clinical signs of dentinogenesis imperfecta (DI) were observed in this case; however, existing literature indicates that even in the absence of definite DI, subclinical hypomineralization associated with collagen defects can significantly weaken the dentinoenamel junction (DEJ) [1, 3, 10]. This compromised DEJ predisposes the enamel to early shearing, thereby exposing the underlying dentin and facilitating accelerated bacterial penetration toward the pulp chambers [1, 6, 7].

In this case, management of deep carious lesions favored the VPT approach over conventional root canal treatment (RCT). In contrast to classic presentations of DI, which are often associated with pulp chamber obliteration, this patient exhibited open and accessible pulp spaces. However, the presence of open apices in the permanent second molars represented a significant therapeutic challenge. Conventional RCT in immature permanent teeth carries an increased risk of failure due to thin dentinal walls and the absence of a natural apical constriction. These challenges were further compounded by the patient's limited mouth opening, which restricted access for precise endodontic instrumentation in posterior teeth. Consequently, VPT using bioactive materials was selected as a biologically favorable alternative to preserve pulpal vitality, promote apexogenesis, and support the long‐term survival of these immature molars [6, 11].

The widespread eruption failure observed in this patient, involving teeth #13 through #45, is consistent with previously reported impaction rates of up to 33.5% in cohorts of patients with OI [2, 12]. Such eruption disturbances are frequently associated with maxillary hypoplasia and inadequate arch space, thereby necessitating careful and ongoing monitoring of eruption patterns. A conservative, non‐extraction approach was adopted for all impacted teeth [2, 6, 13]. This treatment strategy was primarily selected due to the extreme skeletal fragility characteristic of OI and the substantial risk of iatrogenic mandibular fracture associated with invasive surgical retrieval [2, 14, 15]. Additionally, the radiographic absence of a discernible PDL space raised concern for potential ankylosis, further supporting long‐term clinical and radiographic surveillance as a more advisable alternative to surgical intervention [2, 13].

The radiographic identification of preeruptive intracoronal resorption (PEIR) in impacted teeth #44 and #45 is a clinically significant finding. Its presence indicates that dental tissue breakdown in patients with OI may begin within the alveolar bone, independent of exposure to the oral microbial environment. This observation underscores the role of qualitative Type I collagen defects in compromising the developmental integrity of dental tissues prior to their eruption into the oral cavity [3].

Surgical extraction of the retained primary molar roots was done with meticulous care. Although the patient was not receiving bisphosphonate therapy—thereby minimizing the risk of medication‐related osteonecrosis of the jaw (MRONJ) [8, 9]—a minimally invasive surgical approach was strictly adopted. This precaution was essential to reduce the risk of bone micro‐fractures and delayed or impaired healing associated with the altered biomechanical properties of bone in patients with OI [14, 15].

The decision to perform full‐mouth rehabilitation under GA in this patient was primarily driven by the need to prevent systemic and skeletal complications, rather than serving solely as a behavior‐management strategy. Although severe anxiety and non‐cooperative behavior posed significant challenges for conventional dental treatment [7, 11], several factors related to OI represented more critical challenges.

Significant limited mouth opening, consistent with physical findings reported in some OI cases, substantially restricted clinical access and significantly increased the risk of iatrogenic jaw fractures in a conventional dental chair setting [1, 4, 6]. Furthermore, generalized joint laxity—a hallmark feature of OI—carries a heightened risk of mandibular dislocation during prolonged dental procedures [1, 6]. Consequently, GA provided a controlled and safe operative environment that ensured optimal restorative outcomes while minimizing the risk of secondary physical and psychological trauma to the patient [7, 11].

For posterior teeth in patients with OI, SSCs are widely regarded as the gold standard for full‐coverage restoration. Their use is critical in preventing rapid attrition of brittle enamel and subsequent loss of vertical dimension [6, 7, 16]. When tooth‐colored restorations are indicated, careful selection of adhesive systems is essential, as OI‐affected dentin is characterized by a deficient collagen matrix and increased organic content [3, 6, 12]. Recent evidence supports the use of universal bonding agents containing the 10‐MDP monomer, which demonstrates superior clinical performance through chemical bonding with residual hydroxyapatite and reduced microleakage. These properties help compensate for the compromised structural integrity of OI dentin, thereby enhancing restoration longevity in a substrate where conventional bonding strategies often fail [17, 18].

## 7 Conclusion

OI is an extremely rare disorder, and many affected individuals do not survive to an age at which comprehensive dental treatment of the permanent dentition can be undertaken. Consequently, clinical data and reported experiences regarding dental management of such patients remain limited. In this report, we present a rare case describing comprehensive dental rehabilitation in a patient with OI, incorporating detailed medical and dental considerations.

## Author Contributions

**Pegah Mosannen Mozafari:** conceptualization, project administration, writing – review and editing. **Mahsa Talafi Noghani:** investigation, methodology, project administration, supervision, visualization, writing – original draft. **Iman Kashani:** investigation, writing – review and editing. **Reza Beyragh:** project administration, writing – original draft, writing – review and editing.

## Funding

The authors have nothing to report.

## Ethics Statement

For clinical cases, the local ethics committee considers that the patient's consent is sufficient.

## Consent

Written informed consent was obtained from the patient's caregivers to publish this report in accordance with the journal's patient consent policy.

## Conflicts of Interest

P.M.M. serves as the scientific mentor of Shahab Special Care and Oral Medicine Clinic.

## Data Availability

The clinical pictures and radiographs data that support the findings of this study are included in the article.
